# Supplementary material for: Visual Experience Regulates the Intrinsic Excitability of Visual Cortical Neurons to Maintain Sensory Function
Source: Cell Rep. 2019 Apr 16;27(3):685–689.e4. doi: 10.1016/j.celrep.2019.03.073 (PMC6484778; doi:10.1016/j.celrep.2019.03.073)
Supplement: Document S1. Figure S1 [file mmc1.pdf]

Cell Reports, Volume *27*

Supplemental Information

Visual Experience Regulates the Intrinsic Excitability of Visual Cortical Neurons to Maintain Sensory Function

Alexander P.Y. Brown, Lee Cossell, and Troy W. Margrie

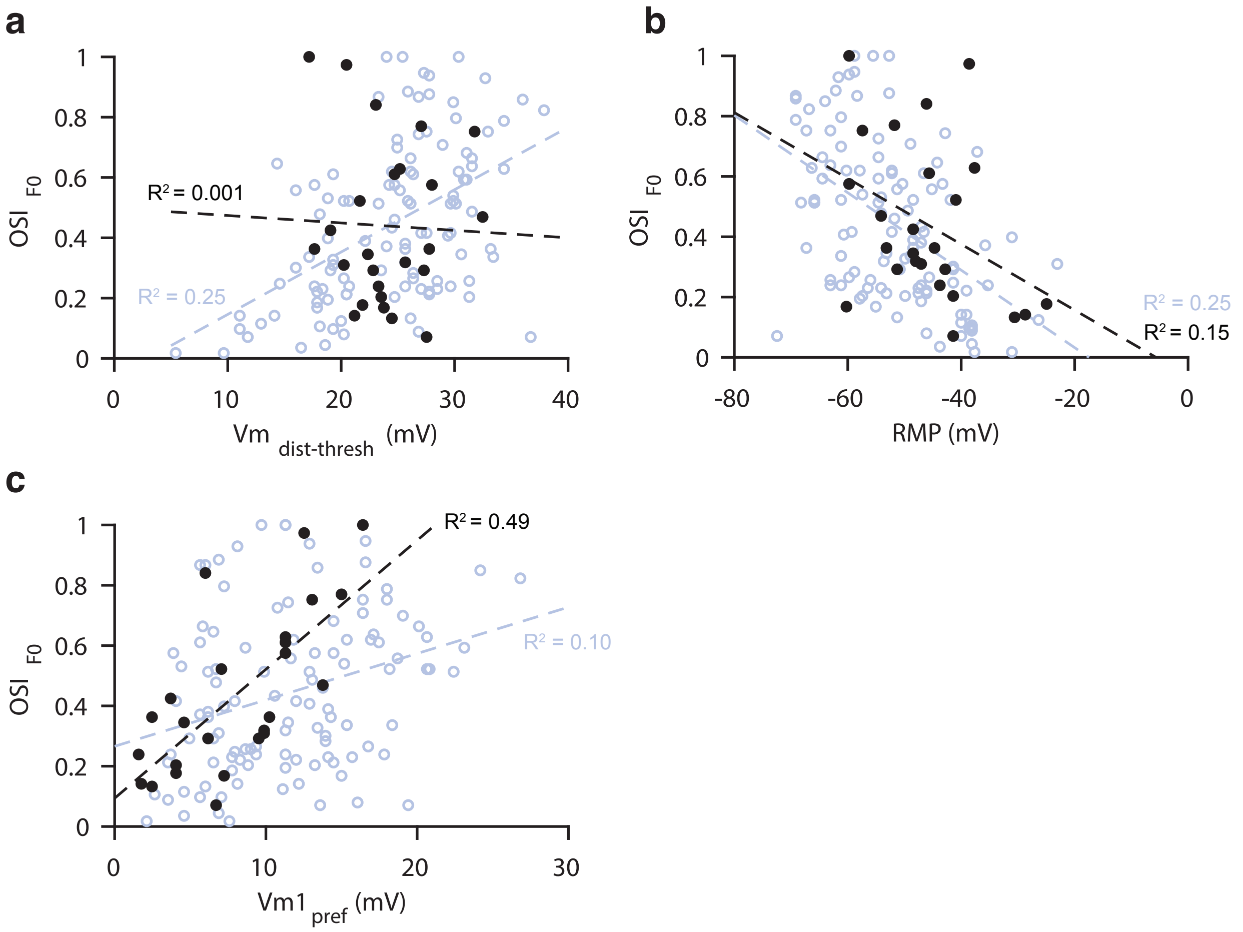

Figure S1. Related to Figure 3. Relationship between intrinsic and synaptically evoked properties and spiking OSI.
Scatter plots showing the correlations between OSI and (**a**) distance between resting membrane potential and spike threshold ($Vm_{dist\text{-}thresh}$), (**b**) resting membrane potential (RMP), and (**c**) the membrane potential modulation at the preferred direction ($Vm1_{pref}$). Data obtained from control (n = 111, blue) and visually deprived (n = 25, black) are overlaid with the line of best fit for each. R^2 shown for control and deprived.
